# Two-dimensional calibration-free odds design for phase I drug-combination trials

**DOI:** 10.3389/fonc.2023.1294258

**Published:** 2023-11-29

**Authors:** Wenliang Wang, Huaqing Jin, Yan Dora Zhang, Guosheng Yin

**Affiliations:** ^1^ Department of Statistics and Actuarial Science, The University of Hong Kong, Hong Kong, Hong Kong SAR, China; ^2^ Department of Radiology and Biomedical Imaging, University of California, San Francisco, San Francisco, CA, United States; ^3^ Department of Mathematics, Imperial College London, London, United Kingdom

**Keywords:** dose finding, drug combination, maximum tolerated dose, non-parametric method, phase I trial design

## Abstract

In oncology, it is commonplace to treat patients with a combination of drugs that deliver different effects from different disease-curing or cancer-elimination perspectives. Such drug combinations can often achieve higher efficacy in comparison with single-drug treatment due to synergy or non-overlapping toxicity. Due to the small sample size, there is a growing need for efficient designs for phase I clinical trials, especially for drug-combination trials. In the existing experimental design for phase I drug-combination trials, most of the proposed methods are parametric and model-based, either requiring tuning parameters or prior knowledge of the drug toxicity probabilities. We propose a two-dimensional calibration-free odds (2dCFO) design for drug-combination trials, which utilizes not only the current dose information but also that from all the neighborhood doses (i.e., along the left, right, up and down directions). In contrast to interval-based designs which only use the current dose information, the 2dCFO is more efficient and makes more accurate decisions because of its additional leverage over richer resources of neighborhood data. Because our design makes decisions completely based on odds ratios, it does not rely upon any dose–toxicity curve assumption. The simulations show that the 2dCFO delivers satisfactory performances in terms of accuracy and efficiency as well as demonstrating great robustness due to its non-parametric or model-free nature. More importantly, the 2dCFO only requires the minimal specification of the target toxicity probability, which greatly eases the design process from the clinicians’ aspects.

## 1 Introduction

The main objective of a phase I clinical trial is to find the maximum tolerated dose (MTD), which is defined as the highest dose with an acceptable probability of dose-limiting toxicity (DLT). For safety reasons, a phase I clinical trial typically assigns one cohort at a time to the most suitable dose level according to some criterion, and the choice of which dose to treat the next cohort is made based on the observed toxicity outcomes of the previous cohorts. Traditional phase I clinical trial designs are mainly for single-drug treatments, such as the 3 + 3 design (1), the continual reassessment method (CRM) (2), the dose escalation with overdose control design (3), the Bayesian optimal interval (BOIN) design (4), and the non-parametric overdose control (NOC) design (5).

In the past decades, combination therapies have demonstrated advantages of higher efficacy, lower toxicity, and fewer side effects compared to monotherapy (6), and thus an efficient design for drug-combination trials is more desirable. A common assumption for phase I clinical trials is the monotonic relationship between dose levels and toxicity probabilities. For single-drug trials, this simply restricts the sequence of toxicity probabilities with ascending dose levels are completely ordered, i.e., higher dose levels induce more severe toxicities. However, for two-drug combinations, this becomes a partial ordering constraint on each row and each column in the two-dimensional toxicity probability space, while the toxicity relationship on the diagonal lines is often unknown, thus imposing new challenges for dose-finding and toxicity probability estimation.

Numerous methods have been proposed for identifying the MTD in drug-combination trials, and most of the existing methods rely on parametric models. Thall et al. (7) proposed a two-stage Bayesian design with a six-parameter model, requiring informative priors based on historical dose–toxicity data from previous single-agent studies on each of the two drugs. Wang and Ivanova (8) proposed a three-parameter Bayesian design using the parsimonious working model for the dose–toxicity relationship. Yin and Yuan (9, 10) developed a joint toxicity probability model for the binary outcomes through a copula-type regression. Wages et al. (11) proposed a partial ordering continual reassessment method (POCRM) for two-dimensional drug-combination trials. Riviere et al. (12, 13) considered a Bayesian design using the standard logistic regression, which still requires priors of toxicity probabilities of the two drugs respectively.

All the aforementioned methods are parametric, requiring either specification of tuning parameters or prior knowledge of the drug toxicities. Due to their parametric nature, these methods often achieve high accuracy in selecting the MTD when precise information is given, at the cost of robustness when such information is missing or inadequate. On the other hand, non-parametric methods tend to be more robust compared to parametric ones. Mander and Sweeting (14) proposed a product of independent beta probabilities design based on conjugate Bayesian inference. The escalation scheme is constructed by estimating a maximum tolerated contour using the posterior probabilities. Lin and Yin (15) developed a two-dimensional Bayesian optimal interval (2dBOIN) design for drug-combination trials by extending the one-dimensional BOIN design to a two-dimensional space. Similarly, the 2dBOIN design makes decisions based on the posterior probability that a given dose’s toxicity probability falls in the pre-specified optimal interval. Razaee et al. (16) introduced a non-parametric Bayesian method for dual agents with truncated beta priors, in which the joint posterior probability of DLT is estimated using a weighted Gibbs sampler.

In this work, we extend the calibration-free odds (CFO) design (17) to two-dimensional drug-combination trials, named the 2dCFO design. Similar to the CFO design, our 2dCFO design is also a non-parametric or curve-free method using purely the odds based on the estimated posterior probabilities. The main advantages of the CFO design are that it requires minimal parameter tuning due to its model-free and calibration-free nature, and furthermore it does not require prior information. The CFO design has shown great advantages in single-drug trials with satisfactory accuracy and robustness properties, and so does the 2dCFO design.

The rest of the paper is organized as follows. In Section 2, we present the methodology and decision rules in the 2dCFO design while reviewing the idea of the one-dimensional CFO design. In Section 3, both fixed and random scenarios are experimented in the simulations to study the operating characteristics of our 2dCFO design in comparison with other state-of-the-art designs. In Section 4, we provide a real trial application to illustrate the operating characteristic of our design by redesigning a drug-combination trial using real data. Section 5 concludes with some remarks.

## 2 Methodology

### 2.1 The 2dCFO design

In a drug-combination trial, a two-dimensional joint toxicity probability space is considered. Suppose that we study the combined toxicity of two drugs, drug *A* and drug *B*, with *J* and *K* dose levels respectively. Let *p_jk_* denote the joint DLT rate for dose combination (*j, k*), *j* = 1*,…,J*, *k* = 1*,…,K*. Let *ϕ* be the pre-specified target DLT rate. Our aim is to find the MTD, which is the dose level with the DLT rate closest to the target *ϕ*,


$$\begin{array}{ccc} {\left(j^{*},k^{*}\right)}_{\text{MTD}}=\underset{j,k}{\arg\min}|p_{jk}-\phi |, & 1\leq j\leq J, & 1\leq k\leq K. \end{array}$$

**Figure 1:**
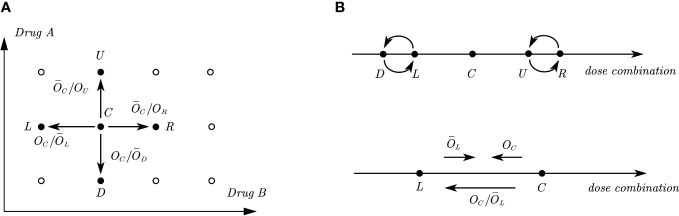
Illustrations of odds and odds ratios in a two-dimensional dose combination space and its one-dimensional interpretations. **(A)** Illustration of a two-dimensional dose combination space, where *C.* is the current dose combination and *L,R,D,U* are the four adjacent dose combinations, where the directions of odds ratios presented in the figure indicate the tendency of dose movement. **(B)** Illustration of one-dimensional interpretations of the two-dimensional dose combination space. The upper figure shows the relative positions of *D,L* and *U,R* with respect to *C.* The lower figure shows the directions of odds and odds ratio between two adjacent doses when the corresponding value is large.

In our design, we enroll cohorts one by one with a fixed cohort size, e.g., the cohort size is 3 by default. After enrollment of *n* cohorts of patients, we obtain the observed toxicity outcomes at all the dose levels as $D_{n}={\left\{\left(x_{jk},m_{jk}\right)\right\}}_{j,k=1}^{J,K}$ , where *x_jk_* and *m_jk_* are the observed number of DLTs and the total number of patients treated at dose combination (*j, k*). Assume that dose level (*j, k*) is the current dose combination, which is denoted as *C*. We further denote the four adjacent dose combinations (*j* − 1*, k*), (*j* + 1*, k*), (*j, k* − 1), (*j, k* + 1) as *L*, *R*, *D*, *U* respectively, representing the left, right, down, and up positions relative to the central or current dose *C*. There are five possible decisions to assign the next cohort, either escalate/de-escalate to the dose level at one of the four positions (Left, Right, Up, Down), or treat at the current dose level, as illustrated in **Figure 1A**. For each dose combination *d* ∈ {*L, D, C, U, R*}, we denote the true DLT rate as *p_d_* and the observed toxicity outcomes as (*x_d_, m_d_*).

The 2dCFO design is constructed based on joint decisions of multiple one-dimensional CFO analyses. Following the partial ordering constraints, it is known that dose combinations *L* and *D* have a lower DLT rate than *C* and dose combinations *U* and *R* have a higher DLT rate than *C*. Reformulating in a one-dimensional space, *D* and *L* are located on the left of *C*, while *U* and *R* are located on the right. However, the relative positions of *D* and *L*, as well as *U* and *R* are not known or specified, as illustrated in **Figure 1B**. From the information above, we can identify four one-dimensional dose sequences with ascending DLT rates: {*L, C, R*}, {*L, C, U*}, {*D, C, R*}, {*D, C, U*}. Thus, the decision for escalating/de-escalating/staying in a two-dimensional space can be made based on the joint decisions of one-dimensional CFO analyses on the four dose sequences. We use the dose sequence {*L, C, R*} as an example to illustrate how we make decisions in a one-dimensional CFO design, and the same method can be applied to the other three dose sequences respectively.

The CFO design uses odds to measure the tendency for escalating/de-escalating the dose level, which is defined as


$$O_{d}=\frac{\mathrm{Pr}\left(p_{d}>\phi |x_{d},m_{d}\right)}{\mathrm{Pr}\left(p_{d}\leq \phi |x_{d},m_{d}\right)},d\in \left\{L,D,C,U,R\right\}.$$

Let $\bar{O}$ *_d_* = 1*/O_d_* denote the reciprocal of *O_d_*. Taking the odds of *L* and *C* as an example, a large value of *O_C_* indicates the current dose combination is too toxic, which suggests de-escalating to the next lower dose combination. Similarly, a large value of $\bar{O}$ *_L_* indicates that the DLT rate of the left dose combination is too low, and thus suggests dose escalation. This is analogous to a battle between the left and current doses: the former tries to push the dose up while the latter tries to push it down. Hence, the ratio *O_C_/* $\bar{O}$ *_L_* measures the tendency or strength for de-escalating to the left adjacent dose level *L*, as shown in **Figure 1B**. That is, a large value of *O_C_/* $\bar{O}$ *_L_* tends to push the dose down. Similarly, *O_C_/* $\bar{O}$ *_D_* measures the tendency or strength to de-escalate to dose combination *D*, and $\bar{O}$ *_C_/O_R_*, $\bar{O}$ *_C_/O_U_* measures the tendency or strength to escalate to dose combinations *R* and *U* respectively, as illustrated in **Figure 1A**.

A non-informative prior Beta(*ϕ*, 1 − *ϕ*) is adopted for each DLT rate *p_d_*. Using the monotonic relationships that *p_L_* < *p_C_< p_R_*, the marginal posterior density functions for *p_L_* and *p_C_* from the left side can be derived as


$$\begin{array}{l} f_{L}(p_{L}|x_{L},x_{C})\propto \;f_{\beta }(p_{L};a_{L},b_{L})\int_{p_{L}}^{1}f_{\beta }(p_{C};a_{C},b_{C})\text{d}p_{C},\\ f_{C}(p_{C}|x_{L},x_{C})\propto \;f_{\beta }(p_{C};a_{C},b_{C})\int_{0}^{p_{C}}f_{\beta }(p_{L};a_{L},b_{L})\text{d}p_{L}. \end{array}$$

where *f_β_* (·;*a_d_,b_d_*) is the density function of Beta(*a_d_,b_d_*), with *a_d_* = *ϕ* + *x_d_* and *b_d_* = 1 − *ϕ* + *m_d_*− *x_d_*. Similarly, for computing the odds ratio between *p_C_* and *p_R_* from the right side, we have


$$\begin{array}{l} f_{C}(p_{C}|x_{C},x_{R})\propto \;f_{\beta }(p_{C};a_{C},b_{C})\int_{p_{C}}^{1}f_{\beta }(p_{R};a_{R},b_{R})\text{d}p_{R},\\ f_{R}(p_{R}|x_{C},x_{R})\propto \;f_{\beta }(p_{R};a_{R},b_{R})\int_{0}^{p_{R}}f_{\beta }(p_{C};a_{C},b_{C})\text{d}p_{C}. \end{array}$$

Both *O_C_/* $\bar{O}$ *_L_* and $\bar{O}$ *_C_/O_R_* can be computed using the Gaussian quadrature or the Monte Carlo method. The two odds ratios are used for making decisions. Escalation/de-escalation is more favorable when the odds ratio is relatively large, and staying at the current dose should be considered when the odds ratio is relatively small. The next step is to find appropriate thresholds for the two odds ratios, by exceeding which we will escalate/de-escalate the dose. Denote the true DLT rate of *p_L_* and *p_C_* as *p*_0_ *_L_* and *p*_0_ *_C_* respectively. Consider the probability of incorrect votes or indications *V_L_* (*γ_L_*), i.e., either the computed odds ratio is large (i.e., *O_C_/* $\bar{O}$ *_L_* > *γ_L_*), suggesting de-escalation, but in fact we should stay at the current dose under the condition of (*p*_0_ *_C_* = *ϕ,p*_0_ *L < ϕ*), or the odds ratio is small (i.e., *O_C_/* $\bar{O}$ *_L_* ≤ *γ_L_*) suggesting escalation but the current dose is in fact overly toxic (*p*_0_ *_L_* = *ϕ,p*_0_ *_C_* > *ϕ*). As a result, the left threshold $\gamma_{L}^{*}$ can be defined as the one that minimizes *V_L_* (*γ_L_*):


$$\begin{array}{l} \begin{array}{ccc} \gamma_{L}^{*} & =\arg\underset{\gamma_{L}}{\min} & V_{L}(\gamma_{L}) \end{array}\\ \begin{array}{cc} =\arg\underset{\gamma_{L}}{\min} & \text{Pr (}O_{C}/{\bar{O}}_{L}>\gamma_{L}|p_{0C}=\phi ,p_{0L}<\phi )+\text{Pr (}O_{C}/{\bar{O}}_{L}\leq \gamma_{L}|p_{0L}=\phi ,p_{0C}>\phi ) \end{array}\\ \begin{array}{cc} =\arg\underset{\gamma_{L}}{\min} & \sum_{i=0}^{m_{C}}\sum_{j=0}^{m_{L}}I\text{ (}O_{C}/{\bar{O}}_{L}>\gamma_{L}) \end{array}\text{Pr (}x_{C}=i|p_{0C}=\phi )\text{Pr (}x_{L}=j|p_{0L}<\phi )\\ \begin{array}{cc} + & \sum_{i=0}^{m_{C}}\sum_{j=0}^{m_{L}}I\text{ (}O_{C}/{\bar{O}}_{L}\leq \gamma_{L}) \end{array}\text{Pr (}x_{C}=i|p_{0C}>\phi )\text{Pr (}x_{L}=j|p_{0L}=\phi ), \end{array}$$

where *I* (·) denotes the indicator function. Similarly, denote the true DLT rate of pR as p0R, and then the right threshold is defined as


$$\begin{array}{l} \begin{array}{ccc} \gamma_{R}^{*} & =\arg\underset{\gamma_{R}}{\min} & V_{R}(\gamma_{R}) \end{array}\\ \begin{array}{cc} =\arg\underset{\gamma_{R}}{\min} & \text{Pr (}{\bar{O}}_{C}/O_{R}>\gamma_{R}|p_{0C}=\phi ,p_{0R}>\phi )+\text{Pr (}{\bar{O}}_{C}/O_{R}\leq \gamma_{R}|p_{0R}=\phi ,p_{0C}<\phi ) \end{array}\\ \begin{array}{cc} =\arg\underset{\gamma_{R}}{\min} & \sum_{i=0}^{m_{C}}\sum_{j=0}^{m_{R}}I\text{ (}{\bar{O}}_{C}/O_{R}>\gamma_{R}) \end{array}\text{Pr (}x_{C}=i|p_{0C}=\phi )\text{Pr (}x_{R}=j|p_{0R}>\phi )\\ \begin{array}{cc} + & \sum_{i=0}^{m_{C}}\sum_{j=0}^{m_{R}}I\text{ (}{\bar{O}}_{C}/O_{L}\leq \gamma_{R}) \end{array}\text{Pr (}x_{C}=i|p_{0C}<\phi )\text{Pr (}x_{R}=j|p_{0R}=\phi ). \end{array}$$

Since the trial for each patient is independent, the number of patients with DLTs follows a binomial distribution, thus we have


$$\begin{array}{l} \text{Pr}(x_{C}=i|p_{0C}=\phi )=\left(\begin{array}{c} m_{C}\\ i \end{array}\right)\phi^{i}(1-\phi {)}^{m_{C}-i},\\ \text{Pr}(x_{L}=j|p_{0L}=\phi )=\left(\begin{array}{c} m_{L}\\ j \end{array}\right)\phi^{j}(1-\phi {)}^{m_{L}-j}. \end{array}$$

We further adopt a Uniform(0, ϕ) prior for *p0L* when *p0L< ϕ* and Uniform(*ϕ*,2*ϕ*) prior for *p0C* when *p0C > ϕ*, and then the following probabilities can be computed using the Gaussian quadrature,


$$\begin{array}{l} \text{Pr}(x_{L}=j|p_{0L}<\phi )=\int_{0}^{\phi }\frac{1}{\phi }\left(\begin{array}{c} m_{L}\\ j \end{array}\right)p_{0L}^{j}(1-p_{0L}{)}^{m_{L}-j}\text{d}p_{0L},\\ \text{Pr}(x_{C}=i|p_{0C}>\phi )=\int_{\phi }^{2\phi }\frac{1}{\phi }\left(\begin{array}{c} m_{C}\\ i \end{array}\right)p_{0C}^{i}(1-p_{0C}{)}^{m_{C}-i}\text{d}p_{0C}. \end{array}$$

As a result, *V_L_* (*γ_L_*) is computed, and so is *V_R_* (*γ_R_*). Therefore, the two thresholds $\gamma_{L}^{*}$ and $\gamma_{R}^{*}$ can be obtained, and the decision follows the decision rules in **Table 1**.

**Table 1:** Decision rules for one-dimensional CFO analysis.

	$O_{C}/{\bar{O}}_{L}>\gamma_{L}^{*}$	$O_{C}/{\bar{O}}_{L}\leq \gamma_{L}^{*}$
${\bar{O}}_{C}/O_{R}>\gamma_{R}^{*}$	Stay	Escalate to *R*
${\bar{O}}_{C}/O_{R}\leq \gamma_{R}^{*}$	De-escalate to *L*	Stay

Similar decisions can be obtained from the other three directions {*L,C,U*}, {*D,C,R*} and {*D,C,U*}. Finally, we can proceed to formulate joint decisions.

### 2.2 Decision rules and the algorithm

1. Treat the first cohort at the lowest dose level (1,1) or a pre-specified dose level.

2. Suppose the current cohort is treated at dose level *C*. We apply one-dimensional CFO analysis on both the horizontal direction {*L,C,R*} and the vertical direction {*D,C,U*}. The joint decisions are formulated as follows.

- If analyses along both directions suggest staying, i.e., the joint decision is to stay at the current (central) dose level, the next cohort will be treated at dose level *C*.
- If one direction suggests escalation (or de-escalation) while the other suggests staying, the joint decision is to escalate (or de-escalate) to the corresponding dose. For example, if the decision for {*L,C,R*} is escalation while the decision for {*D,C,U*} is staying, then the next cohort will be treated at dose level *R*.
- If one direction suggests escalation while the other suggests de-escalation, we need to further compare the contradictory direction using one-dimensional CFO analysis. For example, if the decision for {*L,C,R*} is escalation, while the decision for {*D,C,U*} is de-escalation, we further apply one-dimensional CFO analysis on {*D,C,R*}, because the true toxicity order of {*D,C,R*} is also known. We then escalate the dose level to *R* if the decision is escalation, and the dose level will stay at *C* if the decision is staying, and de-escalate to *D* if the decision is de-escalation.
- If both directions suggest escalation, we will escalate to either *U* or *R*, while the most appropriate direction can be chosen by comparing *O_U_* and *O_R_*. Since $\bar{O}$ *_C_/O_R_* measures the tendency of escalating towards *R*, while $\bar{O}$ *_C_/O_U_* measures the tendency of escalating towards *U*, if $\bar{O}$ *_C_/O_R_ >* $\bar{O}$ *_C_/O_U_*, i.e., *O_R_< O_U_*, we treat the next cohort at dose level *R*, otherwise we treat the next cohort at *U*. If the two odds *O_R_* and *O_U_* are the same, we randomly select one dose from the two to treat the next cohort.’
- If both suggest de-escalation, we will de-escalate to either *L* or *D*. Similarly, if *O_C_/* $\bar{O}$ *_L_ > O_C_/* $\bar{O}$ *_D_*, i.e., *O_L_ > O_D_*, we treat the next cohort at dose level *L*, otherwise we treat the next cohort at *D*. If the two odds *O_L_* and *O_D_* are the same, we randomly select one dose from the two to treat the next cohort.

3. Repeat step 2 until the sample size is reached or the early stopping criteria are met.

The 2dCFO decision rules in Step 2 are summarized in **Table 2**.

**Table 2:** Decision rules for the two-dimensional CFO analysis.

Vertical Decisions	Horizontal Decisions		
	Escalate	Stay	De-escalate
Escalate	Escalate to *R* if *O_R_< O_U_*, escalate to *U* if *O_R_* > *O_U_*, and randomly select *R* or *U* if *O_R_* = *O_U_*	Escalate to *U*	Next dose is decided by 1dCFO analysis on {*L,C,U*}
Stay	Escalate to *R*	Stay	De-escalate to *L*
De-escalate	Next dose is decided by 1dCFO analysis on {*D,C,R*}	De-escalate to *D*	De-escalate to *L* if *O_L_* > *O_D_*, de-escalate to *D* if *O_L_< O_D_*, and randomly select *L* or *D* if *O_L_* = *O_D_*

### 2.3 Early stopping and final selection of the MTD

In practice, it is preferable to impose some early stopping and overdose control strategies to alleviate safety concerns. During the implementation of the 2dCFO, we will terminate the trial if the lowest dose level (1,1) is overly toxic, as determined by Pr(*p*_11_ > *ϕ* | *x*_11_ *,m*_11_ ≥ 3) > 0.95. To further prevent assigning too many cohorts to the overly toxic doses, we exclude dose level (*j*_0_ *,k*_0_) and all the higher dose levels, i.e., (*j,k*), where *j* ≥ *j*_0_ and *k* ≥ *k*_0_ if Pr(*p _j_*_0_ *k*_0_ > *ϕ* | *x _j_*_0_ *k*_0_ *,m _j_*_0_ *k*_0_ ≥ 3) > 0.95.

Upon finishing treating all cohorts, we obtain the cumulative data for all dose combinations as $D_{n}={\left\{\left(x_{jk},m_{jk}\right)\right\}}_{j,k=1}^{J,K}$ . For each dose combination (*j,k*), the estimated DLT rate $\hat{p}$ *_jk_* can be computed as *x_jk_/m_jk_*. To conform with the partial ordering constraints, we further perform a bivariate isotonic regression (18) on the estimated DLT rates $\hat{p}$ *_jk_* using the pool-adjacent-violators algorithm (PAVA). Let $\tilde{p}$ *_jk_* denote the estimator corresponding to $\hat{p}$ *_jk_* in the isotonic regression, and then the MTD (*j* ^∗^ *,k* ^∗^) is selected as


$$\begin{array}{ccc} {\left(j^{*},k^{*}\right)}_{\text{MTD}}=\underset{j,k}{\arg\min}|{\tilde{p}}_{jk}-\phi |, & \text{ where }1\leq j\leq J, & 1\leq k\leq K. \end{array}$$

## 3 Simulation study

### 3.1 Fixed-scenario simulation

To assess the performance of the proposed method, we compare our 2dCFO design with three competitive methods: the two-dimensional Bayesian optimal interval design (2dBOIN), the partial ordering continue reassessment method (POCRM), and the adaptive logistic model design. We conduct simulations on 14 fixed scenarios, in which the number of MTDs varies from 1 to 3 and the target DLT rate is set to be 0.3, as shown in **Table 3**. The 14 fixed scenarios are carefully constructed to encompass all common instances of joint toxicity probability distribution. These scenarios include situations where the MTDs are positioned diagonally and doses are initiated from the minimum dosage and progressively advanced to the maximum dosage. They also incorporate situations where MTDs are sporadically spread across multiple diagonal lines. We set the total number of patients to be 60 with a cohort size of 3. Under each scenario, we run 5000 independent simulations. For the 2dBOIN method, we apply the R package “BOIN” (19) and adopt default values for all parameters, i.e., *ϕ*_1 =_ 0.6, *ϕ*_2 =_ 1.4 and *λ* = 0.95 as suggested in the original paper (11). For the POCRM, the provided R package “pocrm” (20) restricts the cohort size to be 1, while we slightly modified the source code to allow the cohort size to be 3. We adopt six orderings with equal prior probabilities, as suggested by Wages (21), while other parameters are set as default values. For the logistic method, we use the R package “dfcomb” (22), with the prior toxicity probabilities set as (0.2, 0.3, 0.4) and (0.1, 0.2, 0.3, 0.4, 0.5) for the two drugs respectively, and all other parameters are set as default values. The logistic method includes a start-up phase by default, in which the dose level will be increased until the first DLT is observed. Such a start-up phase is considered part of the design and is kept in our simulations. In the simulations, we do not adopt any early-stopping rules for each of the methods, and all patients should be treated before the final selection of the MTD.

**Table 3:** Fourteen fixed scenarios of joint toxicity probabilities for simulations, with the target DLT rate *ϕ* = 30% in boldface.

Drug B										
Drug A	1	2	3	4	5	1	2	3	4	5
	Scenario 1					Scenario 2				
**3**	0.15	**0.30**	0.45	0.50	0.60	0.45	0.55	0.60	0.70	0.80
**2**	0.10	0.15	**0.30**	0.45	0.55	**0.30**	0.45	0.50	0.60	0.75
**1**	0.05	0.10	0.15	**0.30**	0.45	0.15	**0.30**	0.45	0.50	0.60
	Scenario 3					Scenario 4				
**3**	0.10	0.15	**0.30**	0.45	0.55	0.50	0.60	0.70	0.80	0.90
**2**	0.07	0.10	0.15	**0.30**	0.45	0.45	0.55	0.65	0.75	0.85
**1**	0.02	0.07	0.10	0.15	**0.30**	**0.30**	0.45	0.60	0.70	0.80
	Scenario 5					Scenario 6				
**3**	0.07	0.09	0.12	0.15	**0.30**	0.15	**0.30**	0.45	0.50	0.60
**2**	0.03	0.05	0.10	0.13	0.15	0.09	0.12	0.15	0.30	0.45
**1**	0.01	0.02	0.08	0.10	0.11	0.05	0.08	0.10	0.13	0.15
	Scenario 7					Scenario 8				
**3**	**0.30**	0.50	0.60	0.65	0.75	0.08	0.15	0.45	0.60	0.80
**2**	0.15	**0.30**	0.45	0.52	0.60	0.05	0.12	**0.30**	0.55	0.70
**1**	0.07	0.10	0.12	0.15	**0.30**	0.02	0.10	0.15	0.50	0.60
	Scenario 9					Scenario 10				
**3**	**0.30**	0.37	0.42	0.47	0.52	0.08	0.10	0.15	**0.30**	0.50
**2**	0.15	**0.30**	0.37	0.43	0.48	0.04	0.07	0.12	0.16	**0.30**
**1**	0.10	0.12	**0.30**	0.40	0.45	0.01	0.03	0.06	0.08	0.10
	Scenario 11					Scenario 12				
**3**	0.50	0.60	0.70	0.80	0.90	**0.30**	0.42	0.52	0.62	0.70
**2**	0.10	**0.30**	0.50	0.70	0.80	0.10	0.20	**0.30**	0.40	0.50
**1**	0.06	0.10	0.15	**0.30**	0.50	0.05	0.12	0.20	**0.30**	0.40
	Scenario 13					Scenario 14				
**3**	0.42	0.52	0.62	0.70	0.80	**0.30**	0.42	0.52	0.70	0.80
**2**	0.20	**0.30**	0.40	0.50	0.67	0.10	0.20	**0.30**	0.50	0.67
**1**	0.12	0.20	**0.30**	0.40	0.60	0.04	0.06	0.08	0.20	**0.30**

We use four statistics to assess the performance of the methods. The percentage of the correct MTD selection, the percentage of patients treated at the MTD, the percentage of patients treated above the MTD, and the percentage of patients with DLT. The first two criteria measure the accuracy and efficiency of the designs respectively, for which the higher the better. The latter two criteria reflect the risk of the trials, and thus are expected to be as low as possible. All four statistics are computed as the ratio of simulations that meet specific conditions to the total number of simulations. For instance, the percentage of the correct MTD selection is determined by dividing the number of simulated trials that accurately select the MTD by the total number of simulations, which is 5000 in our case.

**Figure 2:**
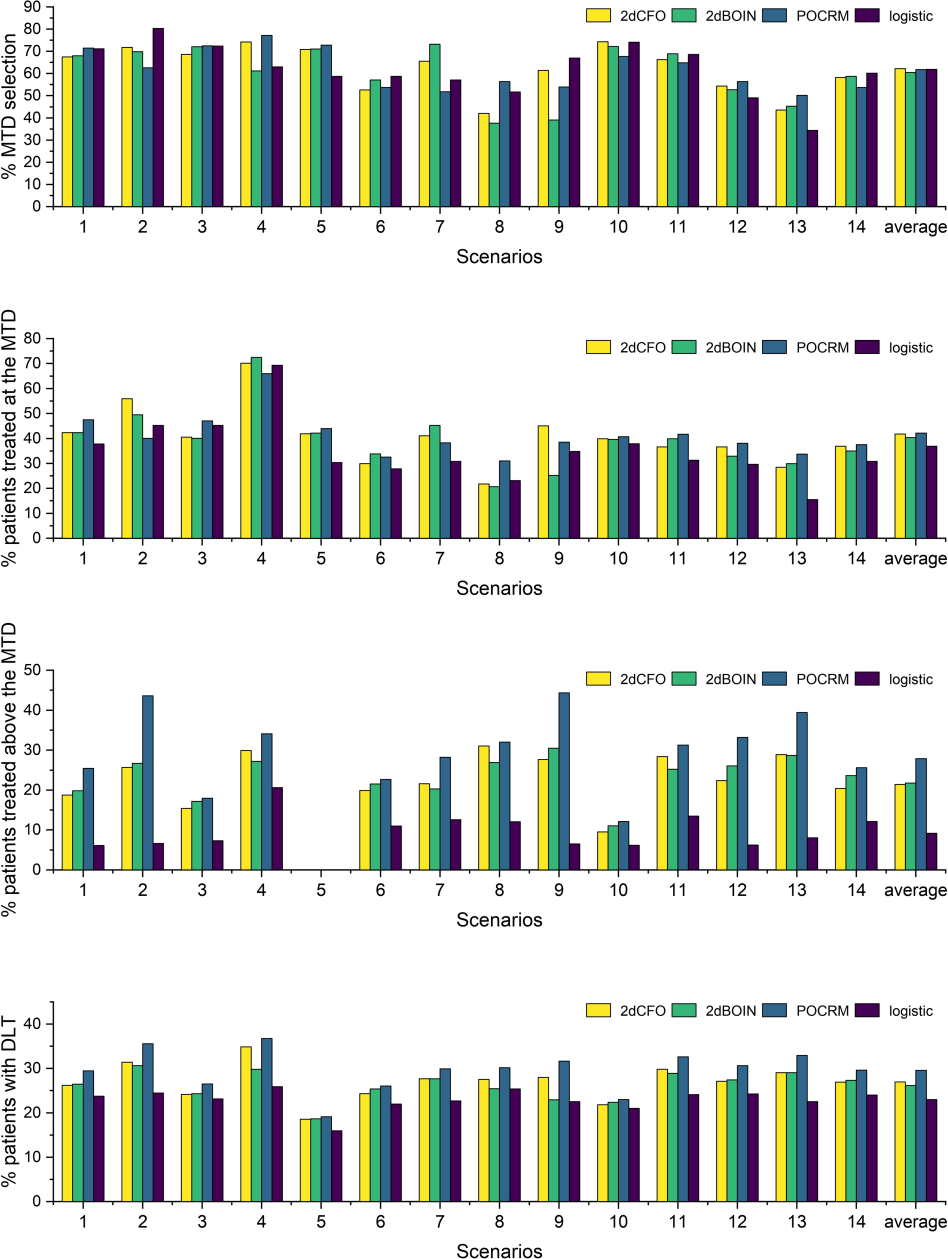
Simulation results under fixed scenarios. The performance of two non-parametric methods (2dCFO and 2dBOIN) and two parametric methods (POCRM and logistic) were compared in terms of the percentage of the correct MTD selection, the percentage of patients treated at the MTD, the percentage of patients treated above the MTD, and the percentage of patients with DLT.

The simulation results for the 14 fixed scenarios are shown in **Figure 2**. According to the results, our 2dCFO design has the highest MTD selection rate (2dCFO: 62.21%, 2dBOIN: 60.48%, POCRM: 61.76%, logistic: 61.88%), and comparable percentages of patients treated at the MTD (2dCFO: 41.78%, 2dBOIN: 40.30%, POCRM: 42.10%, logistic: 36.91%). The safety measures are similar between 2dCFO and 2dBOIN; both have comparable percentages of patients treated above the MTD and percentages of patients with DLT. Although the logistic model has a comparable MTD selection rate, it has a significantly lower percentage of patients allocated to the MTD, indicating a large sacrifice in efficiency. In addition, the logistic model is less robust compared to 2dCFO, as it has a much higher MTD selection rate than other methods in scenarios 2 and 9, but has a much lower selection rate in scenarios 5 and 13. On the other hand, 2dCFO outperforms 2dBOIN with respect to accuracy and efficiency, while keeping similar low risks of toxicity, as indicated by the latter two statistics. Compared with POCRM, 2dCFO has a higher MTD selection rate, a comparable percentage of the MTD allocation, and a significantly lower percentage of patients treated above the MTD and a lower percentage of patients with DLT. Furthermore, we observe that apart from 2dCFO, the other three methods tend to have higher accuracy of MTD selection when MTDs are located at higher dose levels. However, when MTDs are located at lower dose levels, their MTD selection rates fluctuate and are not satisfactory. On the contrary, the 2dCFO design has a consistently high MTD selection rate no matter where the MTDs are located. Scenarios 1–5 and 9–10 have incremental MTDs or MTD contours, and thus the accuracy of the MTD selection across this set of scenarios can reflect the robustness of a method. The results show that our proposed design has consistently high accuracy across all these 7 scenarios. In contrast, the other methods all have significant drops in accuracy in some of the scenarios: The 2dBOIN design has significant drops in accuracy in scenarios 4 and 9. The accuracy of the adaptive logistic design falls in scenarios 4 and 5. The POCRM also has accuracy dropping in scenarios 2, 9, and 10. In conclusion, the 2dCFO design does have satisfactory performance in terms of accuracy, efficiency, and safety, as well as demonstrating high robustness across various patterns of MTD locations.

### 3.2 Sensitivity analysis

**Figure 3:**
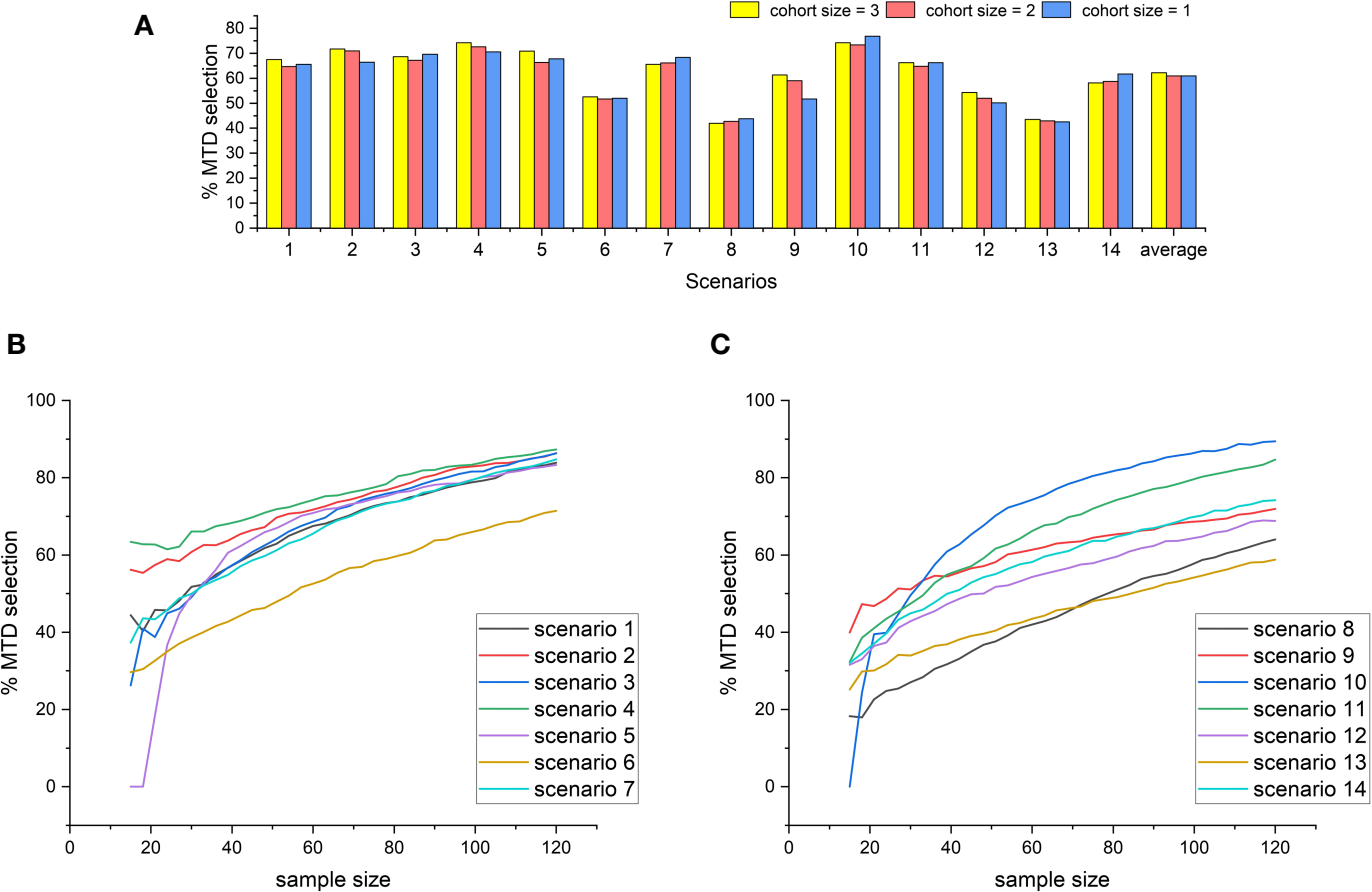
The effect of changes in the cohort size and sample size on the MTD selection rate of the 2dCFO design. **(A)** The MTD selection rate with different cohort sizes. **(B)** The MTD selection rate with increasing sample size(scenarios 1–7). **(C)** The MTD selection rate with increasing sample size(scenarios 8–14).

To study the effect of changes in the sample size and cohort size on the accuracy of the design, we first fix the sample size at 60 while reducing the cohort size from 3 to 2 and 1, with 20, 30, and 60 cohorts respectively. Under each of the settings, we repeat the 14 fixed-scenario simulations and replicate 5000 simulations for each scenario. The results are shown in **Figure 3A**, from which we observe that there are no significant differences in the percentage of the correct MTD selection when the cohort size varies between 1 and 3. However, for most of the scenarios, a cohort size of 3 leads to the highest MTD selection rate, and thus 3 is still the best choice for the cohort size. Furthermore, we study the relationship between the correct MTD selection rate and the sample size. With the cohort size fixed at 3, the number of cohorts increases from 5 to 40, i.e., the sample size extends from 15 to 120. Under each of the configurations, we still repeat the 14 fixed-scenario simulations. The results were shown in **Figures 3B, C**. We can see that there are no significant fluctuations along the curves, indicating the 2dCFO is not sensitive to changes in the number of patients. For each of the scenarios, the percentage of the correct MTD selection keeps increasing steadily as the sample size increases. In some of the scenarios (scenarios 5 and 10), the MTD selection rate starts at a very low percentage due to the limited sample size but increases very quickly as the sample size increases. For most of the scenarios, the MTD selection rate can reach 70 percent at a sample size of 120.

### 3.3 Random-scenario simulation

To further assess the performance and robustness of the proposed method without cherry-picking scenarios, we conduct random-scenario simulations by randomly generating the toxicity probabilities under partial ordering constraints. To generate the random toxicity probabilities, we need to specify the dimension of the matrix *J* × *K*, the number of MTDs with the target toxicity probability *n*_MTD_, and the minimum spacing *ϵ* between adjacent toxicity probabilities. In our simulations, we set the target toxicity probability *ϕ* = 0.3 and *ϵ* = 0.01. We first randomly draw *J* × *K* samples from Uniform(0,1), among which we randomly choose *n*_MTD_ number of elements and set them to be the target *ϕ*. We then check whether the samples have a minimum spacing of *ϵ*; if not, repeat the previous step to re-draw the samples until the minimum spacing constraint is satisfied. The next step is to reshape the sequence of toxicity probabilities into a *J* × *K* matrix and sort the matrix by first sorting each row and then sorting each column of the matrix. Consequently, the resulting matrix satisfies the partial ordering constraints. Lastly, we check whether there are multiple MTDs on the same row or the same column; if so, we go back to the initial stage to redraw the samples and repeat the above steps until at most one MTD is located on each row and each column.

For a better coverage of different dimensions of the toxicity probability matrix, we conduct random simulations under 3 × 5, 3 × 4, 3 × 3 and 2 × 3 cases with a varying number of MTDs, *n*_MTD_. We use the same design parameters as in the fixed-scenario simulations. Under the logistic design, for the case with *J* = 3 and *K* = 4, we set the prior toxicity probabilities of the two drugs as (0.1,0.2,0.3) and (0.1,0.2,0.3,0.4) respectively; for *J* = 3 and *K* = 3, we set the prior toxicity probabilities as (0.1,0.2,0.3) for both drugs; for *J* = 2 and *K* = 3, the prior toxicity probabilities are set as (0.1,0.2) and (0.1,0.2,0.3) respectively. For POCRM, we still adopt the six ordering patterns as in the fixed-scenario simulations. Under each random-scenario setting, we conduct 5000 independent simulations, i.e., randomly generate 5000 probability matrices.

**Figure 4:**
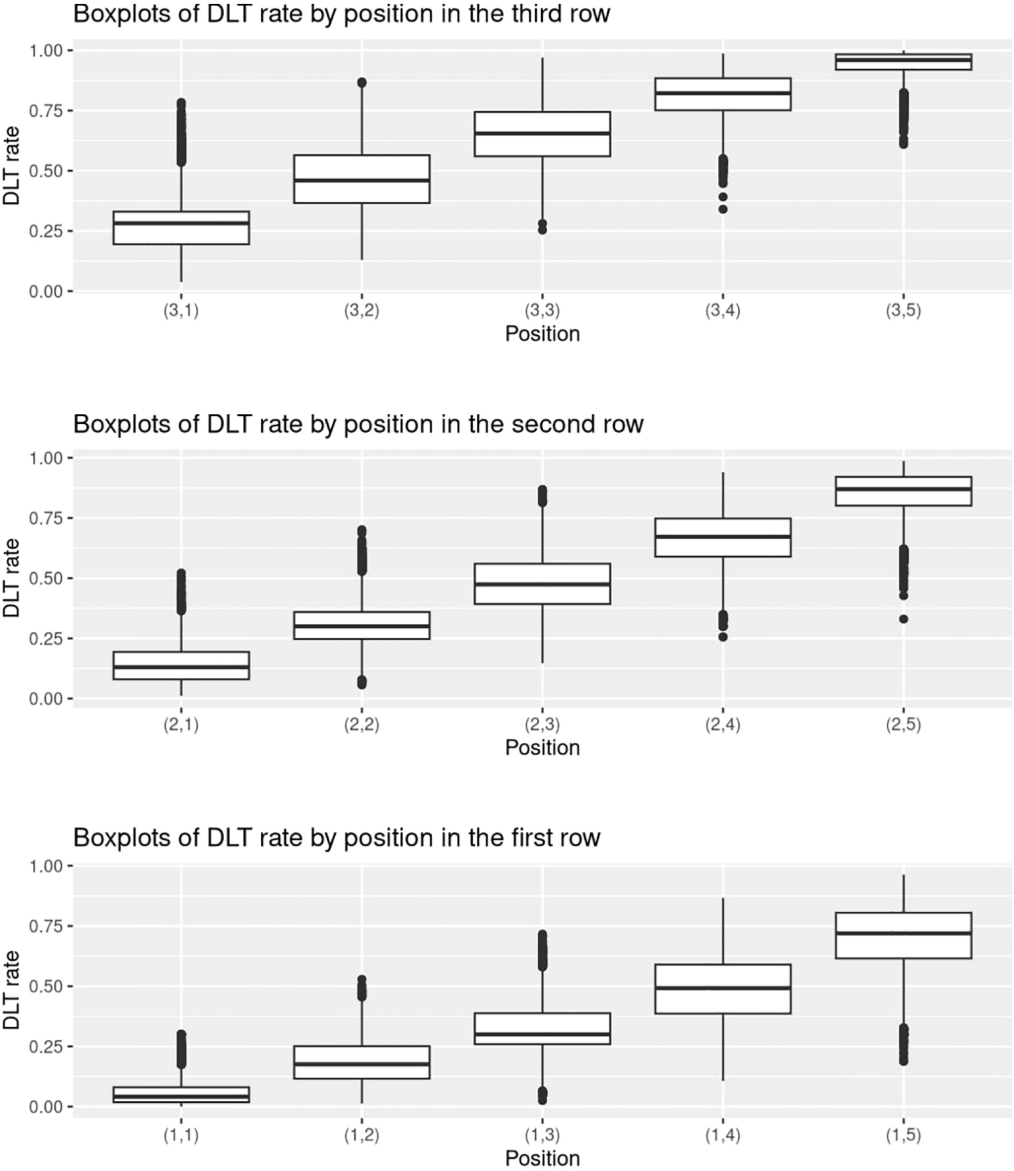
Boxplots of the randomly generated DLT rates in the 5000 simulations under the setting with *J* = 3*,K* = 5*,n*_MTD_ = 1.

**Figure 5:**
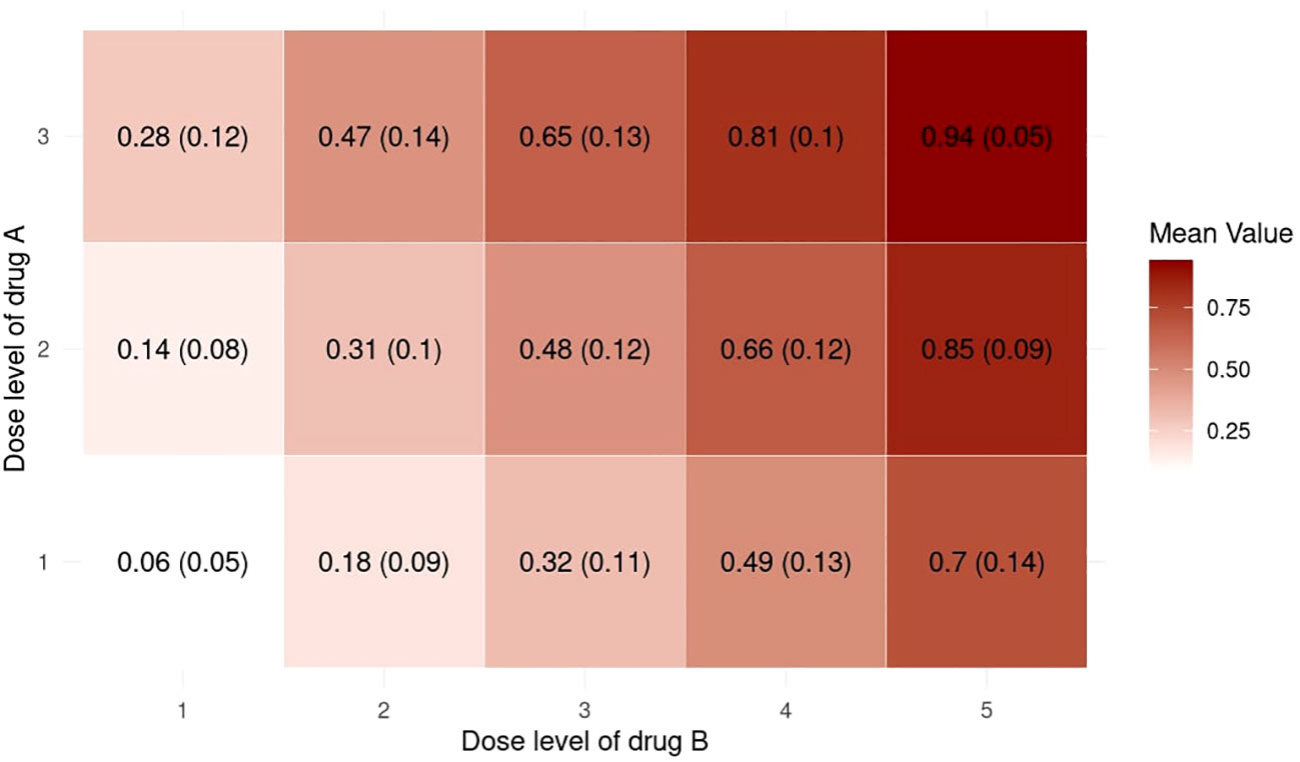
Heatmap of the mean with standard deviation of the randomly generated DLT rates in the 5000 simulations under the setting with *J* = 3*, K* = 5*, n*_MTD_ = 1.

Inspired by Zhou et al. (23), we provide supplementary statistics to better illustrate the characteristics of the randomly generated probability matrices. We use the setting with *J* = 3*,K* = 5*,n*_MTD_ = 1 for illustrative purposes. **Figure 4** shows a collection of boxplots which elucidate the distribution of the 5000 randomly generated DLT rates at each specific dose combination. Furthermore, **Figure 5** displays a heatmap illustrating the mean with standard deviation of the generated DLT rates. As shown by the two figures, the randomly generated DLT rates are well spread out across the matrices, strictly adhering to the partial ordering constraints.

**Figure 6:**
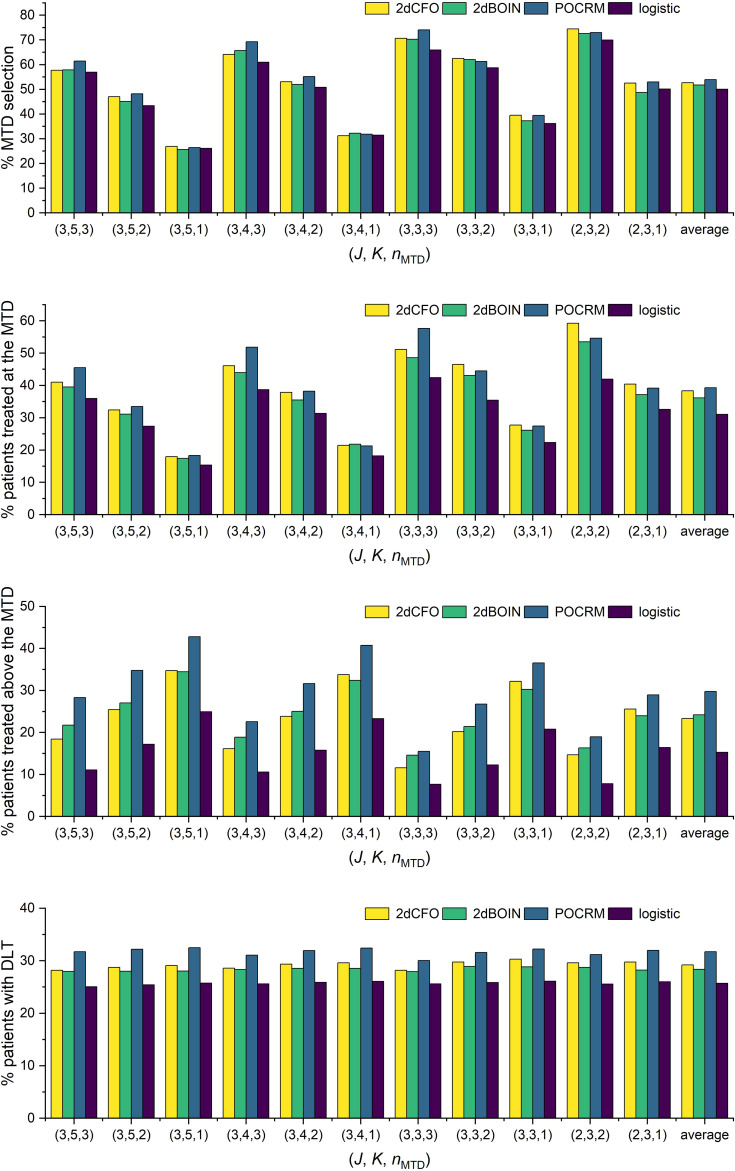
Simulation results under random scenarios. The performances of the four competitive methods were compared under randomly generated toxicity probabilities with *ϕ* = 0.30 and *n*_MTD_ varies.

The simulation results for all these random scenarios are shown in **Figure 6**. In terms of accuracy, 2dCFO has on average a competitively high percentage of correct MTD selection, which is slightly lower than POCRM but higher than the competing non-parametric method 2dBOIN and the logistic method (2dCFO: 52.7%, 2dBOIN: 51.8%, POCRM: 53.9%, logistic: 50.1%). In terms of efficiency, both 2dCFO and POCRM yield significantly higher average percentages of patients treated at the MTD than the other two methods (2dCFO: 38.3%, 2dBOIN: 36.2%, POCRM: 39.3%, logistic: 31.1%). Although POCRM has the best accuracy and efficiency in our random simulations, it has a significantly higher risk in terms of the percentage of patients treated above the MTD (2dCFO: 23.3%, 2dBOIN: 24.2%, POCRM: 29.8%, logistic: 15.2%) and the percentage of patients with DLT (2dCFO: 29.2%, 2dBOIN: 28.4%, POCRM: 31.7%, logistic: 25.7%), when comparing to the other three methods. Furthermore, the risk is particularly higher when there are more dose levels, i.e., in the 3 × 5 and 3 × 4 cases.

When comparing 2dCFO with POCRM, both methods exhibit satisfactory accuracy and efficiency. However, POCRM presents a significantly higher risk than 2dCFO, which is inherited from the CRM itself. Therefore, 2dCFO is more recommended especially when there are few doses under investigation (e.g., the 2×3 case), because the regression model in POCRM may not fit such sparse data well. On the other hand, 2dBOIN has similar performances to 2dCFO due to their non-parametric nature. However, 2dCFO has significantly higher efficiency in assigning the cohorts to the MTD across almost all scenarios. Furthermore, 2dCFO has slightly higher accuracy in selecting the MTD than 2dBOIN, especially in scenarios with few doses (e.g., the 2 × 3 case). The logistic method, on the contrary, has the worst performance in terms of both accuracy and efficiency. Even though it presents significantly lower risk compared to the other three methods, this advantage does not compensate for its shortcomings in accuracy and efficiency.

## 4 Real trial application

To gain more insights into the detailed implementation and application of our proposed method, we consider a real phase I dose-escalation study of Neratinib in combination with Temsirolimus, in patients with advanced solid tumors (24). The study aimed to find the MTD combination of Neratinib and Temsirolimus, which is defined as the highest tolerable dose combination achieving a target DLT rate of less than 0.33. A total of 60 patients were enrolled in the study and each patient received one of 16 combinations of Neratinib {120,160,200,240 mg} and Temsirolimus {15,25,50,75 mg}. The patients were treated in cohorts of size 2 following a bidirectional four-by-four dosing plan, with two initial cohorts treated at the dose combinations (160 mg of Neratinib + 15 mg of Temsirolimus) and (120 mg of Neratinib + 25 mg of Temsirolimus) respectively. The subsequent doses were determined by a non-parametric up-and-down design (25). The toxicity results were shown in **Table 4**. Based on the observed data, we fit a logistic regression model with the doses of Neratinib and Temsirolimus and their interaction term as covariates (15), which gives the estimated DLT rates of all 16 dose combinations in **Table 5**.

**Table 4:** The observed toxicity outcomes and the number of patients treated at each dose combination in the trial of Neratinib and Temsirolimus.

Neratinib (mg)	Temsirolimus (mg)			
	15	25	50	75
240	(2 DLTs, 4 patients)	–	–	–
200	(0 DLTs, 4 patients)	(1 DLTs, 8 patients)	(1 DLTs, 2 patients)	–
160	(1 DLTs, 4 patients)	(1 DLTs, 4 patients)	(0 DLTs, 5 patients)	–
120	(0 DLTs, 2 patients)	(0 DLTs, 4 patients)	(1 DLTs, 5 patients)	(0 DLTs, 4 patients)

**Table 5:** The DLT rates estimated based on the observed toxicity outcomes in the trial of Neratinib and Temsirolimus, where the MTDs are assumed to have a DLT rate of 0.33.

Dose level of Neratinib	Dose level of Temsirolimu			
	1	2	3	4
4	0.24	**0.33**	0.56	0.77
3	0.14	0.19	**0.33**	0.55
2	0.08	0.10	0.17	0.22
1	0.04	0.05	0.07	0.10

The bold values (0.33) are the MTD.

**Figure 7:**
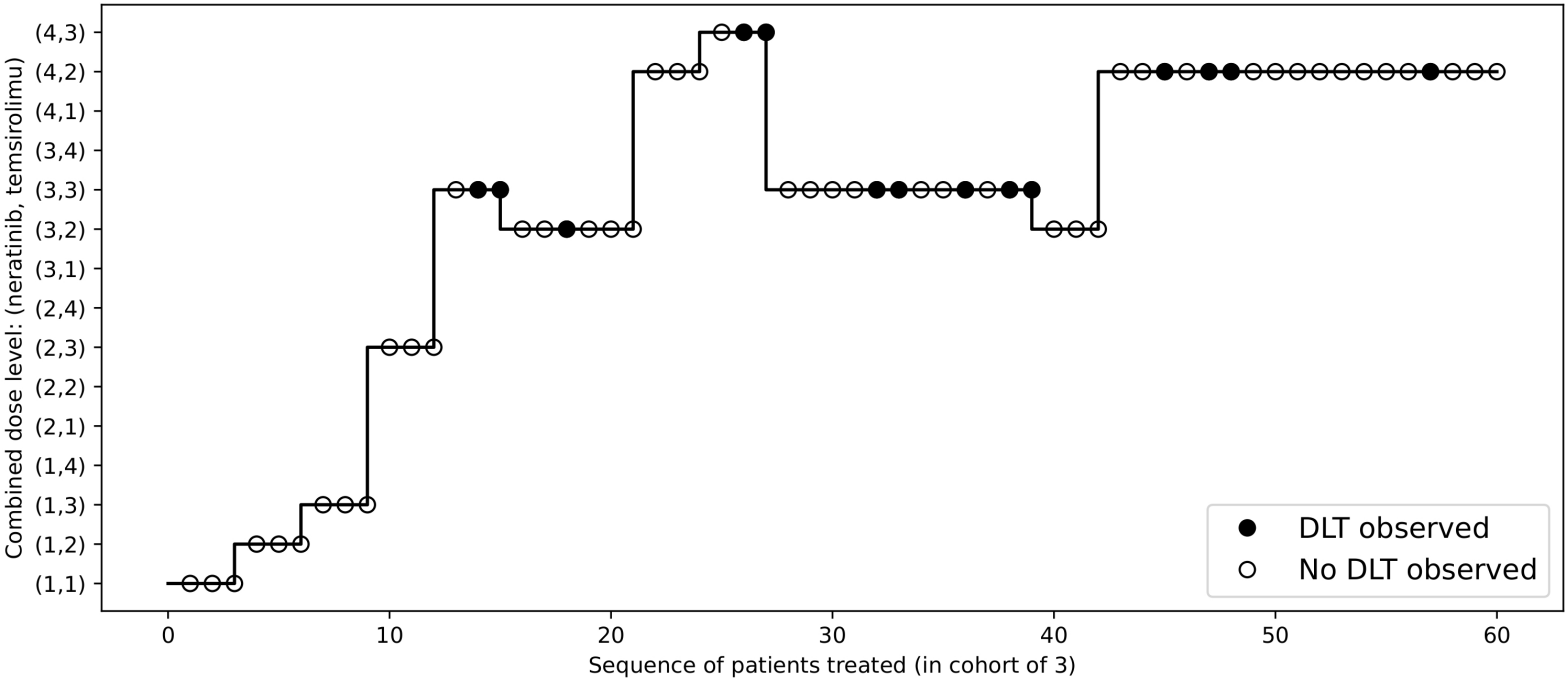
Dose allocations and toxicity outcomes of the redesigned Neratinib and Temsirolimus trial.

To redesign the trial with 2dCFO, we set *ϕ* = 0.33, with a sample size of 60 and a cohort size of 3. Early stopping and overdose control are incorporated in the trial design. The first cohort is treated at the lowest dose level (1, 1), and the subsequent doses are determined according to our proposed decision rules. The dose escalation path and toxicity outcomes are shown in **Figure 7** and the implementation and computation details are given in **Table 6**. Based on the simulated data, we obtain the estimated DTL rates as shown in **Table 7**. The MTD can be selected as either dose level (4, 2) or (3, 3), as both have DLT rates closest to the target 0.33. From the results, we can see that 2dCFO adopts an efficient as well as safe escalation strategy, with 60% (12 out of 20) of the cohorts treated at the two MTDs, only one cohort treated above the MTD, and 23% (14 out of 60) of the patients experienced DLTs. In the simulation of the trial, there is no overdose identified, as Pr(*p_jk_* > *ϕ* | *x_jk_,m_jk_* ≥ 3)< 0.95 is satisfied for all dose combinations. However, the cut-off probability of 0.95 can be adjusted in practice for a more strict or moderate safety rule.

**Table 6:** The implementation details of the 2dCFO design in simulating the Neratinib and Temsirolimus trial, where Pr (overdose)=Pr (*p_jk_* > *ϕ* ∣ *x_jk_*, *m_jk_* ≥ 3) and overdose is indicated by Pr (overdose) > 0.95.

Cohort Index	Dose Level	#DLT	Pr(overdose)	(*O_C_*/ $\bar{O}$ *_L_*, $\gamma_{L}^{*}$ )	( $\bar{O}$ *_C_*/*O_R_*, $\gamma_{R}^{*}$ )	Horizontal Decision	(*O_C_*/ $\bar{O}$ *_D_*, $\gamma_{D}^{*}$ )	( $\bar{O}$ *_C_*/*O_U_*, $\gamma_{U}^{*}$ )	Vertical Decision	Joint Decision
1	(1,1)	0	0.051	–	(9.234,0.127)	Right	–	(9.234,0.127)	Up	Right
2	(1,2)	0	0.051	(0.001,0.473)	(9.234,0.127)	Right	–	(9.234,0.127)	Up	Right
3	(1,3)	0	0.051	(0.001,0.473)	(9.234,0.127)	Right	–	(9.234,0.127)	Up	Up
4	(2,3)	0	0.051	(0.004,0.220)	(9.234,0.127)	Right	(0.001,0.473)	(9.234,0.127)	Up	Up
5	(3,3)	2	0.840	(4.966,0.220)	(0.003,0.127)	Left	(0.473,0.473)	(0.003,0.127)	Stay	Left
6	(3,2)	1	0.441	(0.220,0.220)	(0.071,0.951)	Stay	(0.220,0.220)	(0.127,0.127)	Stay	Stay
7	(3,2)	0	0.153	(0.009,0.135)	(0.466,0.592)	Stay	(0.009,0.135)	(1.076,0.082)	Up	Up
8	(4,2)	0	0.051	(0.003,0.220)	(9.234,0.127)	Right	(0.012,0.190)	–	Stay	Right
9	(4,3)	2	0.840	(0.473,0.473)	(0.003,0.127)	Stay	(299.3,0.473)	–	Down	Down
10	(3,3)	0	0.460	(0.131,0.273)	(0.082,0.082)	Stay	(0.024,0.184)	(0.051,0.592)	Stay	Stay
11	(3,3)	2	0.730	(0.517,0.517)	(0.008,0.484)	Stay	(0.116,0.439)	(0.006,0.475)	Stay	Stay
12	(3,3)	1	0.705	(0.379,0.604)	(0.008,0.300)	Stay	(0.086,0.553)	(0.007,2.081)	Stay	Stay
13	(3,3)	2	0.845	(1.039,0.662)	(0.001,0.215)	Left	(0.248,0.626)	(0.001,1.555)	Stay	Left
14	(3,2)	0	0.051	(0.001,0.103)	(3.288,1.409)	Right	(0.001,0.103)	(363.2,0.475)	Up	Up
15	(4,2)	1	0.153	(0.009,0.135)	(0.466,0.592)	Stay	(0.004,0.665)	–	Stay	Stay
16	(4,2)	2	0.469	(0.103,0.103)	(0.043,0.475)	Stay	(0.030,0.657)	–	Stay	Stay
17	(4,2)	0	0.245	(0.014,0.427)	(0.180,2.081)	Stay	(0.005,0.418)	–	Stay	Stay
18	(4,2)	0	0.115	(0.002,0.319)	(0.580,1.555)	Stay	(0.001,0.539)	–	Stay	Stay
19	(4,2)	1	0.140	(0.003,0.255)	(0.413,1.235)	Stay	(0.001,0.614)	–	Stay	Stay
20	(4,2)	0	0.067	(0.001,0.213)	(1.110,1.110)	Stay	(0.000,0.670)	–	Stay	Stay

Absent values of the odds ratio and thresholds occur at the boundary doses.

**Table 7:** The estimated DLT rate $\hat{p}$ *_jk_* ( $\tilde{p}$ *_jk_*) for each dose combination, where $\hat{p}$ *_jk_* = *x_jk_/m_jk_* and $\tilde{p}$ *_jk_* is the DLT rate estimated by the bivariate isotonic regression.

Dose level of Neratinib	Dose level of Temsirolimus			
	1	2	3	4
4	–	0.19 (0.19)	0.67 (0.66)	–
3	–	0.11 (0.12)	0.47 (0.47)	–
2	–	–	0.00 (0.05)	–
1	0.00 (0.02)	0.00 (0.02)	0.00 (0.02)	–

## 5 Concluding remarks

We propose a two-dimensional calibration-free odds (2dCFO) design for drug-combination trials, which is a major expansion of the one-dimensional CFO design for a single agent. The method relies solely on data-driven approaches, and due to its non-parametric nature, no prior probabilities and specifications of tuning parameters are required. We also do not include any start-up phase or preliminary stage in our simulations. Extensive simulations demonstrate that our proposed method has comparable accuracy, efficiency, and low risk compared to the state-of-the-art non-parametric method 2dBOIN and other parametric methods. The 2dCFO design is demonstrated to be robust in minimizing risk and maximizing efficiency. The real trial application shows that our proposed method is readily applicable to a real phase I escalation study of drug combinations. In addition, a preliminary stage and other overdose control strategies can be incorporated according to practical needs, because neither of them is an internal part of the design.

This study primarily showcases the effectiveness of the 2dCFO design for phase I trials involving a combination of two drugs. However, this research can also be expanded to incorporate seamless phase I/II trials and trials involving more than two drugs. Following the CFO design for a seamless phase I/II trial (17), the only difference in extending to a drug combination trial is the selection of the admissible set, which can be readily determined by adhering to the partial ordering constraints. Furthermore, one can modify the 2dCFO design to accommodate trials involving more than two drugs by simply altering the decision rules. For example, a trial involving three drugs would require 1dCFO analysis on three axes within the 3D toxicity probability space, spanning the horizontal (X-axis), vertical (Y-axis), and depth (Z-axis) dimensions. The final decision can then be derived from a majority vote across these three separate CFO analyses.

No overdose control strategies were adopted in both our fixed and random simulations. There are two types of overdose control strategies, early stopping and dose elimination. Early stopping will only be adopted when the lowest dose (1,1) is overly toxic. However, in our fixed scenarios, the highest DLT rate at (1,1) is 0.3. For random scenarios, according to **Figures 4**, **5**, the DLT rate at (1,1) is much lower than 0.3. Hence both scenarios are not applicable for early stopping rules. Dose elimination, on the other hand, will eliminate the dose and all the higher doses if that dose is overly toxic. This was intentionally omitted from our simulations with the aim of focusing on the escalation performance of the methods, particularly their accuracy and efficiency. Implementing dose elimination rules could potentially skew results, as different methods adopt disparate rules, making it challenging to ensure fairness in comparisons. On the other hand, dose elimination is flexible, with the threshold being tunable for each method. Therefore, we proposed that these should not be considered intrinsic to the method itself, but rather, they should be seen as a variable to bear in mind when executing actual clinical trials.

When planning a real trial, the selection of methods plays a crucial role in achieving desired outcomes. According to our simulation results, in terms of balanced performance characteristics, 2dCFO stands out with competitive accuracy, efficiency, and an acceptable level of risk for safety control. This makes it a particularly suitable candidate, especially for trial designs with fewer dose levels. The performances of the non-parametric methods, namely 2dCFO and 2dBOIN, are largely similar. However, while 2dCFO is optimal for scenarios with fewer dose levels, 2dBOIN may offer a viable alternative in situations where computational complexity is a significant consideration, as it is based on simple patients count in the proposed interval. If safety is not the primary concern, POCRM might be a desirable choice due to its superior accuracy and efficiency. However, should safety be a paramount concern, the logistic method could present a more suitable option. These insights should guide the selection of methods in real trial design, always considering the specific requirements and priorities of each case.

## Data availability statement

The original contributions presented in the study are included in the article/supplementary material. Further inquiries can be directed to the corresponding author.

## Author contributions

WW: Conceptualization, Data curation, Formal Analysis, Methodology, Validation, Visualization, Writing – original draft, Writing – review & editing. HJ: Conceptualization, Methodology, Supervision, Writing – review & editing. YZ: Supervision, Writing – review & editing. GY: Conceptualization, Methodology, Project administration, Supervision, Writing – review & editing.
